# Lost in Translation? A Scoping Review on the Reporting of Team-Based Learning Adaptations

**DOI:** 10.5334/pme.3273

**Published:** 2026-11-06

**Authors:** Jennifer Anne Cleland, Anita Pienkowska, Rachel Ellaway

**Affiliations:** 1 Lee Kong Chian School of Medicine, Nanyang Technological University, Singapore, Singapore; 2 Royal College of Surgeons of Edinburgh, UK; 3 Cumming School of Medicine, University of Calgary, Alberta, Canada

## Abstract

**Introduction:**

Team-based learning (TBL) is a popular instructional method within medical education. Much research has compared the efficacy of TBL to other learning approaches. However, little is known about how and why TBL is contextualised and enacted in practice. To address this gap, we asked: How often, in what ways, and for what reasons has TBL been adapted in medical education?

**Methods:**

A scoping review was conducted following the PRISMA-ScR framework. Searches of seven databases were conducted in 2024 and 2026. Articles were included if they were English-language empirical studies examining the application of TBL within medical education. Data was extracted regarding whether TBL had followed Michaelsen’s original sequence of (broadly) Pre-class Preparation, Readiness Assessment (individual and team) and Application Exercises, or had been adapted. If adapted, we examined which elements of TBL had been contextualized along with the rationale for these changes.

**Results:**

Across the 354 articles reviewed, more than half (n = 183) reported contextualized TBL. The number of articles reporting adaptations increased over time, across all contexts. Which elements of TBL had been adapted and in what ways varied widely but the most frequently changed or omitted component was the Application Exercise (49%), followed by Pre-class Preparation (34%, 62/183). Explicit rationales for adaptations were relatively rare (70/183 studies) but aligned with three broad categories: moving TBL online during the COVID-19 pandemic, saving time/workload, and educational efficacy.

**Discussion:**

TBL contextualization was commonplace. While this itself is not problematic, there was little explicit articulation of how and why medical educators have adapted TBL for their own contexts. More explicit and better reporting of the rationale for TBL contextualization should allow scholars to identify and explore context-specific factors such as institutional pressures, cultural influences, educator preferences etc. that shape how TBL is enacted and how this may alter its efficacy and utility.

## Introduction

In making their case for plural problem-based learning (PBL), Frambach and colleagues began by noting that “globalization has facilitated the exchange and sharing of curricula, educational materials, ideas, innovations, technologies, expertise, policies, and standards” (p. 932) [1]. This referred to the fact that educational interventions designed in and for one context are implemented in many other contexts. While there have been concerns regarding the politics of Western-derived forms of practice treated as universally applicable that come from a small number of Western anglophone institutions [2,3], at a more grassroots level it is important to understand why and how educators make decisions regarding the adoption and implementation of specific teaching methods [4,5].

Our specific interest in this respect was team-based learning (TBL). Originating in the work of Michaelsen and colleagues [6], TBL has been taken up in many different disciplines including science, mathematics and engineering [7] with a particular popularity in health professions education [8]. In our earlier work, we found that TBL research in health professions education was dominated by early adopters, including researchers associated with Michaelsen and with the first ten medical programs to implement TBL [9]. We initially assumed that TBL must be therefore generally implemented in line with the original model. However, while mapping research trends and gaps [10], we observed a wide range of variants.

Given the broader observation that we know relatively little about what happens in TBL classrooms across medical education beyond effectiveness (see Yang & Rajalingam [11], Burgess et al. [12] Rotgans et al. [13], Magdalon et al. [14] for exceptions), this makes it even more important to examine which adaptations are most common and the rationale behind them [4,15,16]. To expand on this, TBL might be delivered as planned by the developers or it may be adapted to be fit for purpose and context [17,18,19]. Neither approach – in line with the original or adaptation – is inherently bad or good [16,20], but the choice to contextualize TBL rather than implement the original format is in itself informative – it may tell us something about medical education in general as well in specific context, educators’ priorities and the settings in which they teach.

It is important therefore to understand why TBL has been implemented differently in different places. Previous reviews have reported the proportions of “classic” and “modified” TBL, but have offered little detail on what was changed in the sequence or why [21,22,23]. Without that detail, the categories risk becoming a judgment about adherence to a model, rather than a way to understand how TBL is put into practice. Explicit articulation of the rationale for TBL adaptations would allow to identify and explore context-specific factors such as institutional pressures, cultural influences, and educator preferences that can shape how TBL is enacted. To explore this, our specific research questions were: How often and in what ways is TBL adapted across contexts? What are the rationales provided for different manifestations of TBL?

## Methods

We employed a scoping review methodology to allow us to engage a wide range of studies [24] reporting on the nature of TBL implementations and adaptions in medical education. In doing so, we followed guidance for scoping reviews [25,26] and reported the results following Preferred Reporting Items for Systematic reviews and Meta-Analyses extension for Scoping Reviews (PRISMA-ScR) [27]. As per scoping review methodology, and because we focused on the instructional implementation rather than research scope and excellence, we did not assess the quality of studies [28].

We conducted two searches, one in April 2024 and an update in January 2026 using the same procedure. We used “team-based learning” and health professions education (HPE) professions in our search strategy (a medical librarian was consulted) on the Ovid (Embase, Medline), PubMed, CINAHL, Web of Science, ERIC, and Scopus databases. Keyword searches were on title and abstract only. No restrictions, such as language or dates were applied at this stage. In case of both screening processes (2024 and 2026) we used SR-Accelerator [29] to remove duplicates and conduct title and abstract screening. The inclusion criteria included the use of TBL and evaluation thereof. While we do not report on evaluation/outcomes in this paper, our knowledge of the TBL literature suggested that empirical papers contained most information on the specifics of reporting innovations and adaptations (the operationalization of change), hence focusing on this body of work. Conference proceedings, non-empirical papers (editorials, commentaries, guidelines, etc.), papers in a language different than English, not about TBL, not including medicine were subsequently excluded. Any doubts were resolved through collective article reassessment. Eligibility was double-checked during full-text screening, resulting in exclusion of mainly non-empirical studies – for details see Figure 1. After the update a final total of 354 records was used for extraction.

**Figure 1:**
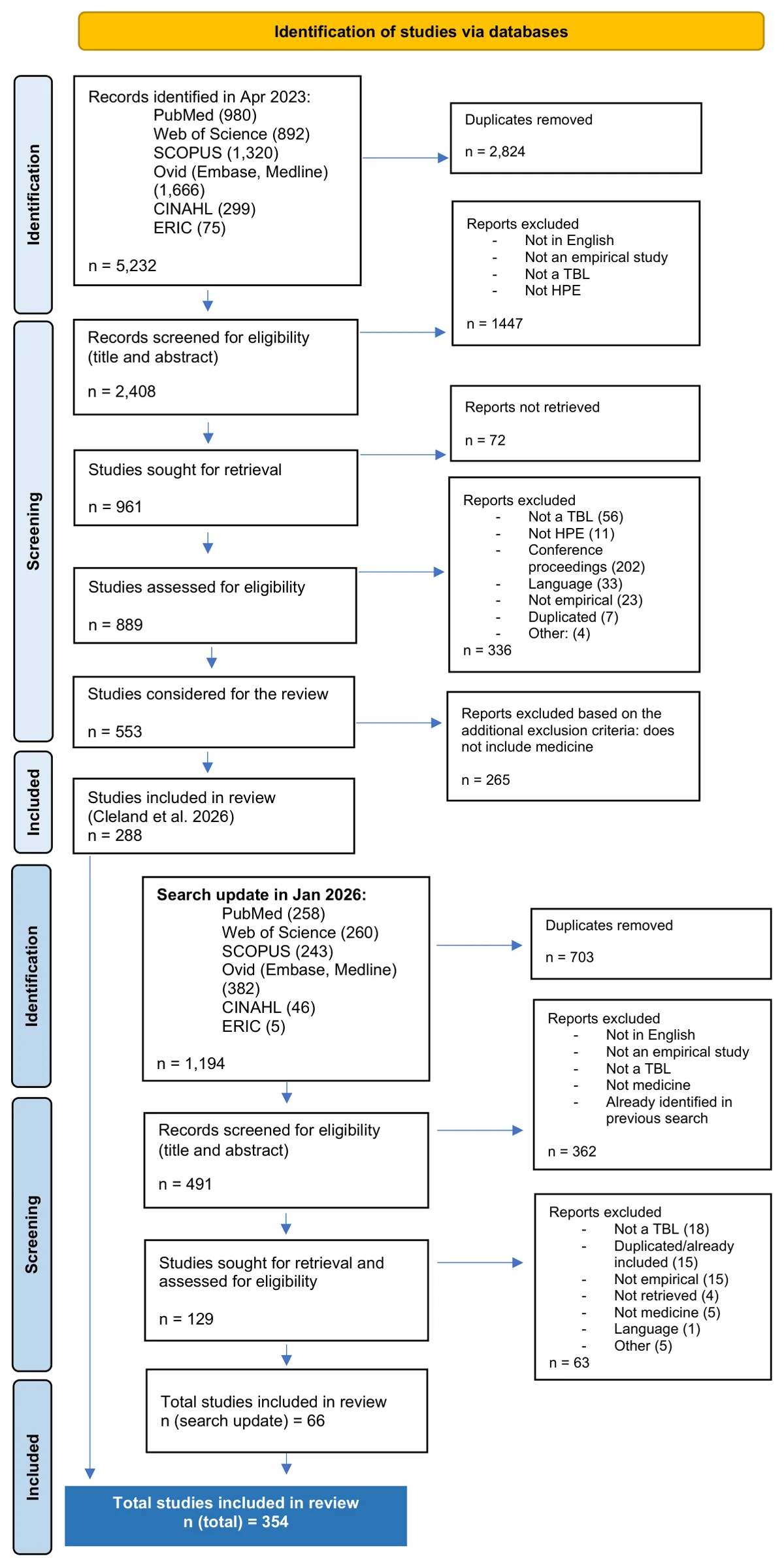
PRISMA-ScR flow diagram for the review of TBL implementation in medical education.

### Data extraction, analysis and synthesis

We took Michaelsen et al.’s sequence of TBL components [6,30,31,32] as the original model against which TBL implementations could be compared to identify whether and how they had been adapted (see Table 1). Data on TBL components were then extracted into a Microsoft Excel spreadsheet, then refined and supplemented with labels to facilitate quantification through descriptive statistics.

**Table 1:** An overview of the TBL sequence and its core components originally proposed by Michaelson and colleagues [6].


PHASES	TBL COMPONENTS

Pre-class (PRE)	Orientation session with TBL familiarization and team formation activity

	Pre-class individual preparation

In class	Individual readiness assurance (IRA)

	Team readiness assurance (TRA)

	Appeals, burning questions and instructor feedback

	Application exercises (AE)

Post-class	Peer evaluation

	Incentive structure


We labelled studies reporting different approaches to adaptations of any of Michaelsen’s essential steps as ‘adapted TBL’ and documented where one or more of the Michaelsen TBL elements had been omitted or altered and where other elements had been added. We treated fully virtual (online) TBL as one adaptation but also examined what else had been adapted where TBL had been moved online. We then conducted a series of analyses to identify temporal and geographical patterns of adaptation. We report on the spread of countries using TBL and reporting Michaelson or adapted TBL over time. Finally, we extracted and synthesized data through content analysis [33] to record the rationales given for any reported adaptations to the Michaelsen TBL model.

### Researchers’ positionality

No research is immune to the influence of researchers’ perspectives and positionalities, which shape everything from the framing of research questions to the data classification, synthesis, and interpretation [34]. This study was inspired by our earlier work with TBL where we became familiar with the literature as well as how TBL is enacted in our context (JC, AP). These insights led to discussions as to what is, and what is not TBL, questions that we wished to consider in relation to published studies as well as our own empirical research. Throughout the research process, we engaged in ongoing reflexive dialogue about both our positions on TBL (e.g., JC had been Vice-Dean of Education in a medical school with TBL as its core pedagogy, AP contributed to TBL teaching, and RHE brought a theoretical perspective on educational activity designs to bear on the project), the tensions and synergies generated by our disciplinary diversity (psychology, humanities and informatics), our differing theoretical commitments and methodological preferences, and our distinct educational and life trajectories. These negotiated positionalities, articulated diversly through multiple discussions, contributed to both the potential influences on and the rigour of this work.

### Ethical approval

This was a secondary analysis of published research studies, so ethical approval was not required.

## Results

Our analyses showed that 38% (136/354) of articles followed Michaelsen’s TBL in-class sequence of PRE, IRA, TRA and AE, while 52% (183/354) adapted TBL with format (e.g., online) or/and sequence alterations. These are discussed in more detail later. The remaining 10% (35/354) of articles did not provide sufficient information to infer what their implementation had involved.

We found that adaptations of TBL in medical education occurred since the early days of its use – our review identified the first report of modified TBL in 2008 – but the number of adapted TBL in the articles reviewed increased over time (see Figure 2).

**Figure 2:**
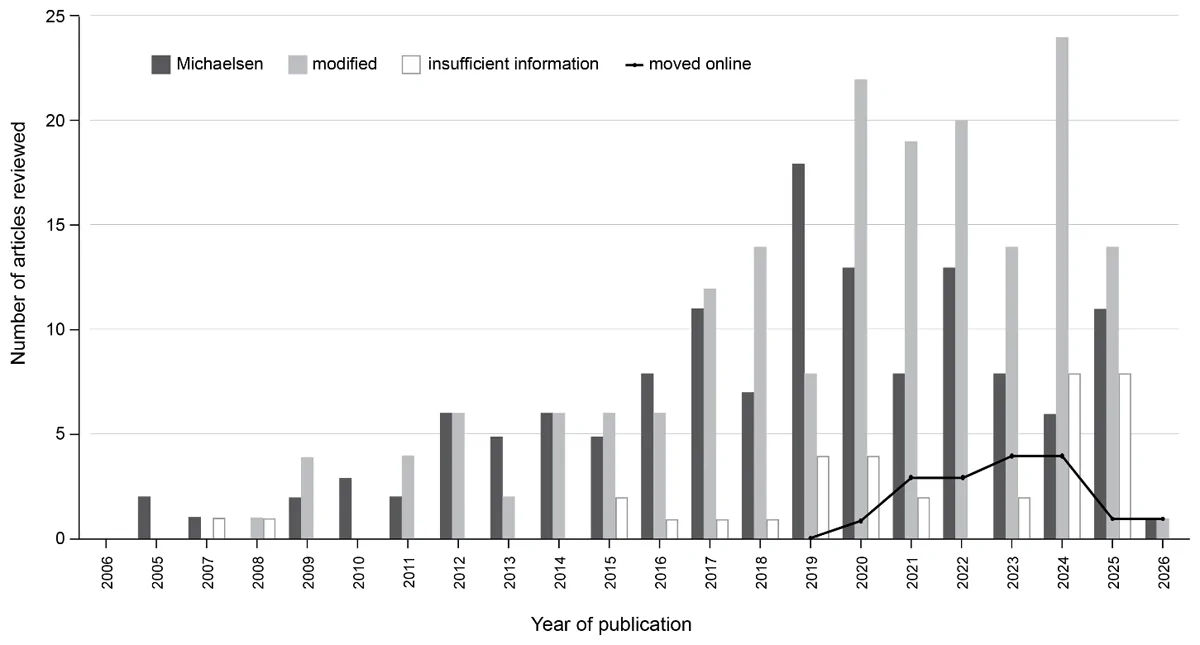
Numbers of articles published by year describing adherence to the Michaelsen model, adaptations to this model, those with insufficient reporting, and those describing moving TBL online.

Notably, the adaptation reported in 32/183 (18% of those categorised as modified) articles was moving TBL completely online. Most of these papers were published in the last five years suggesting that much of this particular adaptation was associated with the COVID-19 pandemic and the resulting transition from in-person to online teaching.

A geographical analysis showed that in most regions adaptations were more common than implementations in line with Michaelsen model of TBL, other than African where the numbers of articles were very small and the Eastern Mediterranean where there was a balance of both implementation approaches – see Figure 3. The ratio of TBL in line with Michaelsen to adaptations was the same for all four regions reporting more adaptations than adherence to the original Michaelsen model.

**Figure 3:**
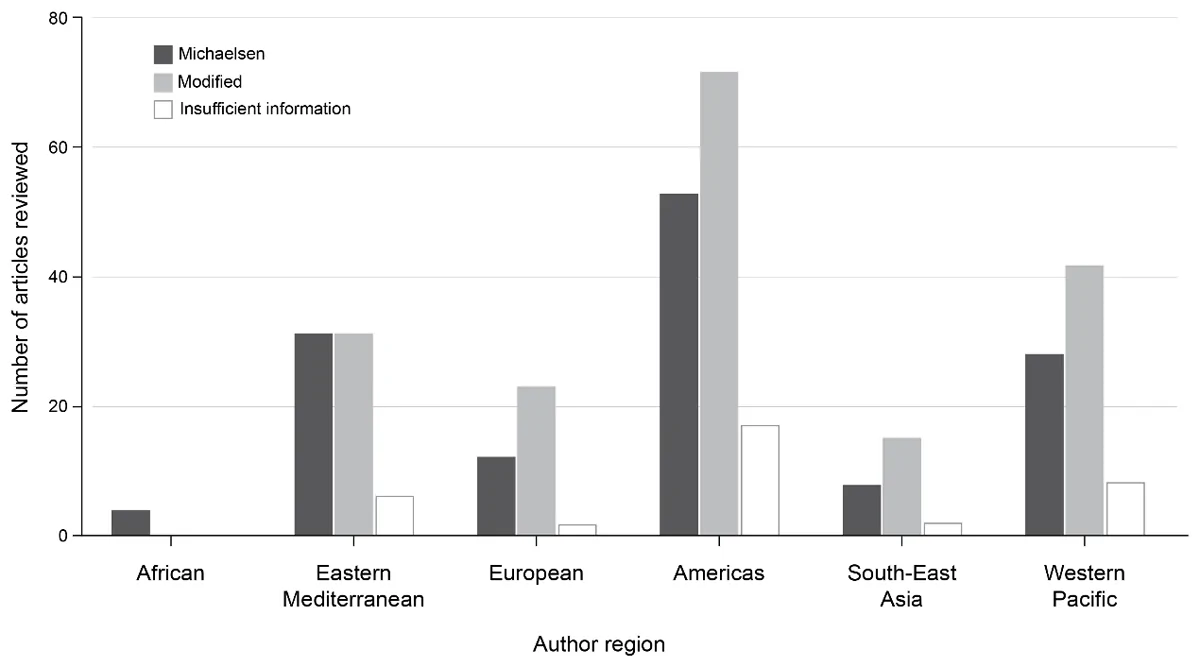
Frequency of articles by region classified by Michaelsen and modified TBL approaches.

### The extent and nature of TBL adaptations

We present these results in the order of most likely to be adapted within Michaelsen’s sequence: AE, PRE, TRA, IRA, elements added and fully virtual implementation. The most frequently changed or omitted component within Michaelsen’s sequence was the AE (49%, 89/183), followed by pre-class preparation (34%, 62/183), TRA (20%, 37/183), and IRA (19%, 35/183).

Application exercise (AE) adaptations most often involved either complete removal of the AE (42%, 37/89) or format changes (58%, 52/89), such as incorporating open-ended questions [35,36,37,38,39,40,41,42,43,44], student presentations [38,45,46,47,48,49,50,51,52], simulations [53,54,55,56,57,58], hands-on practice [59,60,61,62,63,64], gallery walk [35,43,65,66,67], role-play [40,56,68,69] and virtual patients [70,71].

Changes to pre-class preparation (PRE) included its removal (29%, 18/62) or a format change (71%, 44/62), including treating prior classes as preparatory work [63,72,73,74,75,76,77,78,79,80,81,82,83], adding a task [39,49,68,84,85,86,87,88,89,90,91,92], moving IRA to before class thus impacting PRE format [39,63,74,88,92,93,94,95,96,97] or making the pre-class preparation collaborative [62,63,89,98,99,100].

Similarly, changes to TRA included its removal (62%, 23/37) or a format change (35%, 13/37) such as making its questions sequential [54], having different questions than IRA [63,97] and using concept maps [101]. Some changes to TRA format impacted IRA’s format change, such as using practical or application-based exercises while concurrently removing AEs [102,103,104,105,106], or open-ended questions instead of choice-based [107], including using script concordance tests [108]. Finally, changes to IRA included its complete removal (40%, 14/35) and format adaptations (57%, 20/35) such as including on-the-spot questioning by the facilitator [53].

More than a third of articles reviewed reported adding new components (34%, 62/183), most often in the form of a formal lecture (56%, 35/62) while others had added tests and practical exams [39,49,109,110,111,112,113,114], group reviews and discussion [115,116,117,118], student presentations [48,86,119], simulation and role-play [76,114,120,121] and demonstrations [63,100].

Slightly more than half of the articles we reviewed (56%, 98/183) reported just one adaptation, including changing AEs (34%, 33/98) or adding a new element to the sequence (30%, 29/98). The remaining (49%, 89/183) reported two or more adaptations (48 articles reported two, 30 articles reported three, and the remaining eight articles reported four or more adaptations). Among the latter, the most extensively modified implementation involved removing the IRA and TRA components entirely, reshaping PRE into a task-oriented activity with a pre-TBL test, altering the AE component to focus on discussion of preparation cases, and adding a post-TBL quiz [110].

### Rationale for TBL adaptation

Explicit rationales for adapting TBL were provided in just 70 of 183 (38%) studies using an adapted approach. Where a rationale for adaptations was provided, these typically fell into three broad categories. The first (and simplest) involved moving TBL online during the COVID-19 pandemic [14,122,123,124] and was largely about translating the Michaelsen model to a different medium rather than altering the model *per se*.

A second category reflected attempts to make TBL more efficient or to fit available time. Sometimes this involved removing or adapting certain components of TBL to shorten classes or reduce faculty workload. For example, Chitkara et al. [125] adapted TBL to “shorten formalized learning sessions and allow for more time in the clinical learning environment” (p.2). Cabray et al. [95] changed the readiness assurance process (IRA and TRA) to an exercise which could be done outside class time so that “more of [it] to be used throughout the curriculum” (p. 2). Smeby et al. [126] reduced the two-step readiness assurance phase to “a quick warm-up exercise” (p. 286) for time-saving reasons. Jansen & Cavdar [127] adapted TBL to save both time and faculty workload while Luitjes et al. [128] simplified many aspects of TBL “to minimize administrative burden” (p. 5). Another efficiency-oriented adaptation involved adding a lecture into the TBL sequence [129,130,131,132], with the hope that this would increase learner readiness or engagement [133]. Other papers described changing or removing parts of the TBL sequence because they did not work in their context. For example, Vasan and colleagues stated that “although classical TBL requires inter-team discussion our students and faculty found it to be a redundant exercise; hence we discontinued this phase after the pilot year” (p. 335) [91].

A third category of educational rationales focused on making TBL more interactive and engaging, often by adding other educational activities. For example, Cusick [134] added a serious game exercise to promote small-group discussion in a large classroom setting. Both Pek et al. [58] and Luetmer et al. [63] added anatomy demonstrations and simulations, while others merged case-based (AE) and team-based (TRA) learning activities [63,106,135,136].

Independent of the kinds of adaptations reported we were unable to identify from the rationales provided whether TBL was adapted from the outset (i.e., when it was first introduced in a particular place) or whether it was modified at a later date, nor whether adaptations had been made at a particular point in time or over an extended period of time.

## Discussion

Most TBL research in medical education has focused on whether its application realized the intended goals, typically in terms of student outcomes or perceptions [10], while relatively little effort has gone into examining what TBL implementations look like in practice. Our study sought to address this gap in the literature by examining how TBL is adapted, and the reasons provided for such contextualisation.

We found that, when first introduced to medical education, TBL was implemented mostly in line with the original non-contextualized model by Michaelsen [30,31,32]. However, over time, as the use of TBL spread, more TBL adaptations were reported to the extent that now more than half of the whole body of literature reports what we refer to as contextualised TBL. Adaptation is widespread, with no obvious pattern by region. The kinds of contextualizations (what was adapted, to what extent, and in what ways) varied widely although certain elements were more often removed (e.g., the application exercise, pre-class preparation) or retained (e.g., individual and team testing) than others. Explicit rationales for adaptations were relatively rare but tended to align with three broad categories: moving TBL online during the COVID-19 pandemic, saving time and/or reducing workload, and improving educational efficacy.

Adaptation is not in itself problematic. Indeed, both the wider education literature [4,16] as well as the medical education literature [1,5,15,137,138] posit that curricular/instructional procedures often vary, and indeed should vary, because of factors such as local policies, norms and constraints. However, the issue is that less than half of the papers in our review provided a rationale for contextualizing TBL (and even then, often lacked critical details). This means that, as we found, it is impossible in many articles to determine whether adaptations reflected pragmatic considerations, pedagogical intentions, cultural attitudes and norms, contextual constraints, or other factors.

Drilling down to specifics, we do not know why certain elements of TBL tend to be retained during contextualization, while others are more likely to be altered or removed. Without sufficient information, it is difficult to know how and why authors created their particular version of TBL, and, in turn, without this clarity it is difficult to identify transferable practices which might be helpful to those considering the use of TBL in their own contexts. More explicit articulation of the rationale for TBL contextualization is essential to identify and explore context-specific factors that impact on how TBL is enacted. Prospective case studies with medical schools or educators considering the implementation of TBL might provide insight into why TBL is considered the best approach for the particular context, as well as what influences the particular version of TBL which is ultimately developed/enacted.

It also seemed to us that TBL has often been used as an fairly broad “umbrella” term to describe instructional approaches which have some aspects of the original non-contextualized TBL model proposed by Michaelsen and colleagues [30,31,32], but have been highly customized. This is not an issue in itself but is problematic for the field if claims about TBL efficacy are being based on studies of very diverse versions of TBL [10,139]. This point, about potentially comparing ‘apples and oranges’, is relevant in respect of claims about other widely circulating educational innovations such as competency-based medical education, programmatic assessment, flipped classroom, OSCEs, problem-based learning, and simulation (to name but a few). In other words, the need to articulate how and why educational interventions are adapted is a more general point. To support this, we offer some simple guidance in the form of CONTEXT — Contextualizing Educational Interventions: a Checklist for Transparent Adaptation (see Box 1).

**Box 1 CONTEXT — Contextualizing Educational Interventions: a Checklist for Transparent Adaptation**

1. Make clear whether the intervention was based on a reference model. If so, then cite the specific model. If no reference model was used, then describe why this was the case.
2. Describe what the implemented intervention involved and make clear what adaptations were made (e.g. what was added, removed, replaced, combined, shortened, or reordered) and when the adaptations were made compared to the reference model.
3. Explain why each adaptation was made (e.g. learner needs, curriculum requirements, staffing, time, space, policies, other resources).
4. Describe whether the adapted intervention worked as intended and consider how the adaptations altered the outcomes of the intervention.
5. Make sure that any knowledge claims made in respect of the intervention note the adaptations and show caution in relating them to knowledge claims made in respect of the reference model.

Given the large number of studies identified in our original searches, we had to restrict our focus to medical education. This meant we may have missed relevant discussions about TBL adaptations across the broader spectrum of HPE. We focused on empirical papers only as we were interested in the specifics of reporting implementations that were subsequently evaluated, but appreciate that other forms of dissemination (e.g., commentaries, conference papers) may have also described adaptations. Similarly, our focus on English-language publications may have resulted in potentially overlooking valuable insights from papers published in other languages. We did not drill down in detail as to proportion of contextualized and non-contextualized implementations from individual countries: our more detailed analysis of the geographical range of TBL studies has been previously published [9]. We can say that the diversity of countries is vast, and each country will have its own social, cultural and historical norms which will influence not just TBL but all aspects of medical education [140].

We took care to review manuscripts in full to locate rationales for change (where these were included) and our use of content analysis aligns with scoping review methodology, enhancing credibility [141]. We examined what was explicitly reported rather than what was implied because good reporting of curriculum adaption and enactment is critical to improve rigour and grounding of medical education research and progressing the field of HPE research.

Although we drew on the concepts of implementation (non)contextualization to explain our findings, further work in this space may benefit from more explicit use of theory, to provide more explanatory insights into TBL contextualization.

These limitations notwithstanding, this study provides a substantive exploration of how TBL has been contextualized as well as how often, and with what justification.

## Conclusion

Although it has long been known that educational innovations are often significantly contextualized to fit different implementation contexts, what these adaptations are, why and when they are made, and what led to adaptation has not been systematically studied in TBL. We sought to address this gap and in doing so demonstrated a range of contextualizations but few justifications thereof. This paper thus raises practical and philosophical issues regarding the quality of reporting of, and explanations for, TBL contextualizations, and more general messages for recording and reporting adaptations of any educational intervention.

## Data Availability

Data are available on reasonable request. Researchers who wish to obtain further details may contact the corresponding author with a methodologically sound proposal.

## Patient and Public Involvement

Patients and the public were not involved in the design, conduct, or reporting of this literature review.
